# Association between serum levels of C-reactive protein and personality traits in women

**DOI:** 10.1186/1744-9081-4-16

**Published:** 2008-04-02

**Authors:** Susanne Henningsson, Fariba Baghaei, Roland Rosmond, Göran Holm, Mikael Landén, Henrik Anckarsäter, Agneta Ekman

**Affiliations:** 1 Department of Neuroscience and Physiology, Section of Pharmacology, Göteborg University, P.O. Box 431, SE 405 30 Göteborg, Sweden; 2 Institute of Medicine, Department of Metabolism and Cardiovascular Research, Göteborg University, Göteborg, Sweden; 3 Department of Clinical Neuroscience, Section of Psychiatry S:t Göran, Karolinska Institutet, Stockholm, Sweden; 4 Section of Psychiatry and Neurochemistry, Göteborg University, Göteborg, Sweden

## Abstract

**Background:**

While low-grade inflammation has consistently been observed in subjects with depression, studies on the possible relationship between inflammation and other aspects of brain function are as yet sparse. In this study, we aimed to investigate the possible association between serum levels of the inflammation marker C-reactive protein (CRP) and personality traits.

**Methods:**

In this study, serum levels of high-sensitivity CRP were determined by ELISA in a population of 270 42-year-old women recruited from the population registry who had been assessed using the Temperament and Character Inventory. Self-reported previous or ongoing depression was also recorded. Unpaired two-tailed *t*-tests were used for comparison between two groups and correlations were evaluated by the calculation of Pearson's r-coefficient.

**Results:**

The temperament trait harm avoidance was positively (*r *= 0.227, *p *< 0.05) and the character trait self-directedness was negatively (*r *= -0.261, *p *< 0.01) associated with serum levels of CRP (*p*-values corrected for multiple comparisons). The correlations between the personality traits and CRP were observed also after exclusion of subjects reporting ongoing depression (n = 26). Whereas women reporting ongoing depression showed significantly increased levels of CRP as compared to non-depressed women (n = 155), women reporting a history of depression displayed no significant difference in CRP levels as compared to women that reported that they had never been depressed.

**Conclusion:**

Serum levels of CRP in women was found to be associated with the personality traits harm avoidance and self-directedness. In addition, moderately elevated levels may be a state dependent marker of depression.

## Background

Following the introduction of commercial assays with high sensitivity for C-reactive protein (CRP) moderately elevated levels of this compound in serum have become a well-established marker of low-grade inflammation. The finding that depression is often associated with such an increase in CRP – that has by now been corroborated in several studies [1-4] – is an observation of considerable importance, as is the recent finding that a similar aberration may be observed also in subjects with panic disorder [5]. If this association between CRP and psychiatric morbidity is a consequence of depression and anxiety influencing the immune system, or if low-grade inflammation may contribute to the development of depression or panic disorder, however is as yet unknown. To cast further light on why CRP is associated with depression and anxiety, and to explore the possible relationship between this marker of low-grade inflammation and other aspects of behavior, including personality traits, hence are important tasks.

The relationship between depression and panic disorder on the one hand, and elevated levels of CRP on the other, is rendered even more important given the well-established but as yet unexplained relationship between these two disorders and cardiovascular morbidity [6-14]. The observations that enhanced CRP is an important risk factor for coronary disease [15-21], and that CRP promote atherosclerotic processes and endothelial cell inflammation [22,23], make it tempting to suggest that low-grade inflammation may be a factor of importance for the association between depression/anxiety and cardiovascular disease.

One obvious way of enhancing our insight into the relationship between CRP and psychiatric disorders is to explore to what extent CRP is associated also with other aspects of brain function, including both other psychiatric disorders and normal personality traits. Exploring the possible relationship between personality and CRP is motivated also by the fact that not only depression and panic disorder, but also certain personality traits, have been suggested to enhance the risk for cardiovascular disease [24-27].

To this end, this study was designed to explore the possible relationship between personality traits and CRP. For the assessment of personality, the Temperament and Character Inventory (TCI) [28] was chosen, being one of the most widely used instruments for the assessment of personality in current psychiatric research. The TCI is based on a self-report questionnaire and comprises seven dimensions, two of which have been suggested to render the individual more prone to develop depression – harm avoidance (being high in subjects at risk for depression) and self-directedness (being low in these subjects) [29]. The primary question prompting this study hence was to what extent there is an association between personality traits as assessed using TCI on the one hand, and CRP levels on the other, in non-depressed subjects, our *à priori *hypothesis being that especially those personality traits that have been shown to be associated with an enhanced risk for depression might be associated with elevated CRP. A rough estimate of ongoing or past depression was obtained by means of a brief yes/no questionnaire based on the DSM general criteria of major depression. The study provides preliminary support for the notion that high harm avoidance and low self-directedness are indeed associated with elevated CRP in middle-aged women recruited from the general population and negating ongoing depression.

## Methods

### Subjects

All women born on uneven days in the year of 1956 and living in Göteborg, Sweden, constituted the primary cohort (n = 1137); this population-based cohort had originally been recruited for a study of obesity, anthropometrics, and cardiovascular risk factors [30]. Reported self-measurements of body weight, height, and circumference ratio over the waist and hips (WHR), were completed and returned by 80% of the original cohort. WHR was then used for a selection of 450 women in total with low, median or high WHR. Of these women, 270 (60%) volunteered to provide blood samples for hormone analyses. At the time of blood sampling, collection of questionnaires (TCI questionnaires were returned by 201 women) and an anthropometric examination were performed recording weight, length and WHR. The women also self-reported smoking habits. In order to screen for previous episodes of depression, the women were asked to answer 'yes' or 'no' on the question whether they had previously felt depressed for a two-week period, or if they had experienced loss of interest or pleasure for a two-week period. To screen for ongoing depression, they were asked to answer 'yes' or 'no' on the question if they now did feel depressed or experienced a loss of interest or pleasure, and had been doing so for at least two weeks. At the time of the investigation the participants were 42 years old and displayed a body mass index (BMI) of 24.9 ± 4.4 kg/m^2^. Thirty-four percent were smokers. Women using hormonal contraceptives (20 women out of 201) were excluded due to reported increased levels of CRP in users of such substances [31-33]. Individuals with CRP ≥ 5 mg/L (n = 16) were also excluded since this may be an indicator of an acute inflammatory response (see Determination of CRP). Three women fulfilled both these exclusion criteria. In total, the study regarding personality and CRP thus comprised 168 women. Information regarding self-reported ongoing or past depression was obtained from 216 women and after exclusion of women using hormonal contraceptives and/or with CRP levels ≥ 5 mg/L, 181 women remained to be included in the investigation of a possible association between depression and CRP; 166 of these women had completed the TCI questionnaire and were part of the study regarding serum levels of CRP and personality. All participating women gave their informed consent and the study protocol was approved by the ethical committee of Göteborg University. The trial was carried out according to the Helsinki Declaration.

### Determination of CRP

Venous-blood samples were obtained between 0800 h and 1000 h after an overnight fast in the follicular phase of the menstrual cycle. Serum samples were stored at -80°C until analysis four years later. CRP levels were determined by means of a high-sensitive double-antibody ELISA purchased from Immundiagnostik A, Bensheim, Germany. Women with elevated CRP levels (≥ 5 mg/L) were excluded from further analysis in line with the instructions of the manufacturer of the assay the since such levels may be indicative of an acute inflammatory response [34-36]. The inter-assay and intra-assay coefficients of variance were 6.0% and 0.1–4.6%, respectively.

### Personality assessment

TCI is a psychometric instrument based on a self-administered true/false questionnaire and designed to assess personality along four temperament dimensions referring to individual differences in basic emotional responses: novelty seeking (high scores imply e.g. exploratory behavior and impulsiveness; low scores imply e.g. an indifferent or reflective personality), harm avoidance (high scores imply e.g. pessimistic worry and passive avoidant behavior; low scores imply a relaxed and outgoing personality), reward dependence (high scores imply e.g. social attachment and dependence on approval of others; low scores imply e.g. detachment), and persistence (high scores imply e.g. ambition and diligence; low scores imply an inactive and modest personality). TCI also measures three character dimensions referring to individual differences in goals, values, and self-conscious emotions: self-directedness (high scores imply e.g. responsibility, purposefulness and self-acceptance; low scores imply e.g. immaturity and habits incongruent with long-term goals), cooperativeness (high scores imply e.g. social tolerance and integration in the society; low scores imply e.g. revengefulness and unhelpfulness), and self-transcendence (high scores imply e.g. imagination, wisdom and integration of universe as a whole; low scores imply e.g. impatience and lack of humility) [28,37]. In the present study, a Swedish 238-item translation of the TCI was used [38]. The TCI factors and sub-dimensions were analysed using normative data.

### Statistical analyses

Unpaired two-tailed *t*-tests were used for comparison between two groups and all measured values are expressed as mean ± SD. Correlations were evaluated by the calculation of Pearson's *r-*coefficient; the significance of the correlations were also controlled for the confounding factors BMI and smoking. Due to multiple significance tests, *p*-values were adjusted by means of the Bonferroni correction (*p*-values were multiplied with the number of TCI factors that were assessed). *p*-values < 0.05 were considered statistically significant.

## Results

The mean serum levels of CRP in the women included in the analysis of personality traits (n = 168) was 1.17 ± 1.19 mg/L (median level 0.63 mg/L). The TCI factors harm avoidance and self-directedness correlated significantly with CRP (see Table 1). After adjustment for BMI and smoking, the correlations remained significant; with respect to HA, they were strengthened. The correlations between CRP, on the one hand, and harm avoidance and self-directedness, on the other, remained significant also after exclusion of women self-reporting ongoing depression (data not shown). When comparing women confirming past but not present depression (n = 71) and women without a history of depression (n = 71), harm avoidance did not correlate with serum levels of CRP in either of the groups. Women reporting a history of depression (but denying ongoing depression) showed a significant correlation between self-directedness and CRP levels (*r *= -0.326, *p *< 0.05, corrected for multiple comparisons) whereas the CRP levels of women never being depressed did not significantly correlate with self-directedness (*r *= -0.209, *p *> 0.05). When analysing the sub-dimensions of harm avoidance, fatigability/asthenia displayed the strongest correlation with CRP. With respect to self-directedness, the sub-dimensions purposefulness and congruent second nature correlated significantly with the inflammation marker after corrections for multiple comparisons (see Table 1).

**Table 1:** Correlations between CRP levels and temperament and character dimensions assessed by TCI (n = 168).

	Correlation	*p*-value	Bonferroni corrected *p*-value
Novelty seeking	-0.027	ns	
Harm avoidance	0.227	0.003	0.02
*Anticipatory worry*	*0.149*	*0.05*	
*Fear of uncertainty*	*0.165*	*0.03*	
*Shyness*	*0.185*	*0.02*	
*Fatigability and asthenia*	*0.218*	*0.004*	
Reward dependence	0.076	ns	
Persistence	0.039	ns	
Self-directedness	-0.261	0.0006	0.004
*Responsibility*	*-0.151*	*0.05*	
*Purposefulness*	*-0.278*	*0.0002*	
*Resourcefulness*	*-0.193*	*0.01*	
*Self-acceptance*	*-0.124*	*ns*	
*Congruent second nature*	*-0.213*	*0.006*	
Cooperativeness	-0.020	ns	
Self-transcendence	-0.043	ns	

ns = non significant

Women who reported that they suffered from an ongoing depression (n = 26) displayed significantly higher levels of CRP than women denying being depressed (n = 155): 1.69 ± 1.44 versus 1.14 ± 1.17 mg/L (*p *< 0.05). This difference remained significant also after exclusion of smokers (data not shown). Women confirming past but not present depression (n = 78) did not differ from women without a history of depression (previous or ongoing) (n = 77) regarding CRP levels (1.20 ± 1.13 versus 1.08 ± 1.13 mg/L); similar results were obtained also when analysing smokers and non-smokers separately.

## Discussion

The main finding of this paper is that the temperament trait harm avoidance was positively, and the character trait self-directedness negatively, associated with CRP levels in a population-based cohort of women. In line with this outcome, an inverse correlation between harm avoidance and self-directedness has been observed by others [39], and was confirmed by our data (not shown).

This is the first study examining CRP levels in subjects in which personality traits have been assessed using the TCI. Previous studies regarding personality traits, measured with other instruments, versus CRP levels, also are sparse. Proneness for anger and hostility has however been associated with elevated levels of CRP [4,40-42]. Also, a study regarding fear for terror attacks revealed, in women but not in men, a positive association between fear and elevated CRP levels after adjustment for generalized anxiety, depressive symptoms and other confounding variables [43].

Although the literature is not unanimous [44-46], a large number of studies suggest that ongoing depression is associated with elevated levels of CRP [1-3,47-50]. In line with this notion, women in our study reporting ongoing depression displayed elevated CRP levels. Since previous studies have shown that depression is often associated with high harm avoidance and low self-directedness state [51-54], it might be suggested that the observed association between these personality traits and CRP merely reflects an underlying depression. However, the association between personality traits and CRP was found also in women denying ongoing depression, suggesting that these personality traits *per se *are related to low-grade inflammation, irrespectively of an ongoing depression.

Different explanations for the relationship between high levels of CRP and depression have been put forward, including the possibility that depression influences the immune system, hence eliciting low-grade inflammation, and the alternative explanation that low-grade inflammation may cause depression. The present study, indicating that CRP may be associated with personality traits, assumed to be relatively stable throughout life, casts new light on this issue. Of interest in this context is however the fact that high harm avoidance and low self-directedness, i.e. the two traits displaying significant associations with CRP in this study, are important predictors of depression [29]. One possibility would be that low-grade inflammation may be both the result of actual or perceived stress [55] – and hence associated with certain personality traits – and a cause for depression, subjects with high harm avoidance and low self-directedness being characterized by low-grade inflammation that may trigger depressive episodes.

Notably, previous studies as well as our data suggest that levels of CRP are higher during an ongoing depression than after recovery, hence suggesting CRP to be a state dependent rather than a trait dependent marker of depression [47]. On the other hand, the available data do not exclude the possibility that CRP levels are slightly elevated also after recovery, and between episodes in recurrent depression [1,2,47].

Importantly, twin studies [56] suggest the heritability of CRP levels to be considerable. This might reflect that the underlying determinants of CRP formation such as obesity or proneness to low-grade inflammation are hereditary, but may also be the result of a more direct influence of genes on CRP production. Several reports have thus shown that polymorphisms in the gene coding for CRP are associated with baseline levels of circulating CRP [57,58]. These polymorphisms may hence be of possible importance for the inflammatory response [59]. When discussing possible explanations for the association with personality traits and depressed mood on the one hand, and CRP levels on the other, in terms of causality, the possibility that certain genes may exert a parallel influence on CRP levels and on brain development and function should not be excluded. The fact that genes are important for personality traits, including those assessed by the TCI scale [37], as well as for traits such as aggression and irritability [60], is well established.

Previous studies have suggested that the apparent association between symptoms of depression and CRP may be partly due to a confounding variable, e.g. high BMI [44]. It is hence important to note that the association between CRP and the traits harm avoidance and self-directedness was not weakened by correcting for BMI or smoking. On the other hand, it should be taken into consideration that we did not control for other factors that may influence CRP levels such as diet [61], exercise [62], and socioeconomic status [63]. In order to exclude individuals with an acute infection or inflammation and avoid false high levels of CRP women with CRP levels exceeding 5 mg/L were excluded. However, the acute phase response is a continuum and no true normal range for CRP levels is established. It has been suggested that levels below approximately 3 mg/L represent "normal" values and CRP levels above 10 mg/L reflect a clinically significant inflammatory state [35,64]. Since blood samples were obtained once only, it cannot be excluded that high levels in some subjects were the results of an ongoing infection or inflammation.

CRP being associated with certain personality traits, and, at the same time, being an important risk factor for cardiovascular disease, makes it tempting to suggest that it may serve as a mediator between these two variables. The TCI sub-dimensions that most clearly correlated with CRP, i.e. fatigability/asthenia and purposefulness, may be factors predisposing individuals to burnout, a state characterized by emotional exhaustion and feelings of inadequate control over one's job [for refs, see [65]]. Burnout has been associated with microinflammation [66] as well as with cardiovascular disease [67,68] also after controlling for depression and other confounding variables. Interestingly, CRP has been suggested to directly influence atherosclerosis [22,23]. A speculative interpretation of our study would be that women being confident, optimistic and self-sufficient, by displaying lower levels of CRP, also may exhibit a decreased risk for cardiovascular disease. This suggestion is well in line with previous studies [69-71].

Limitations of this study are that the number of assessed subjects was relatively small, and that the presented associations, though significant, were modest in term of *r*-values. The population was recruited by means of population register, but the fact that the participants had accepted to participate may render a group somewhat non-representative for the general population. A strength of the study is that all studied subjects were of the same gender and age, hence reducing age- and sex-related sources of variability, and that all blood samples taken for analyses of CRP levels were obtained in the same (follicular) phase of the menstrual cycle [72]. At the same time, the homogeneity of the group with respect to sex and age obviously limits the possibility to draw more generalised conclusions from the results, and makes further studies investigating the relationship between levels of CRP and personality traits in women at other ages and in men highly warranted. Another important limitation of the study is that depression was not assessed using a structured interview, but only by means of a few questions. Previous studies however suggest that very brief depression questionnaires, comprising only a couple of questions, such as the one used in this trial, do display an acceptable sensitivity [73,74]; there are hence reasons to believe that the observation of a relationship between personality traits and CRP in the non-depressed group is not largely influenced by the erroneous inclusion of depressed subjects in this analysis. Also, the self-reported rate of depression in this female population is well in line with other reports [75]. This notwithstanding, the results should be regarded as preliminary until replicated in women evaluated by a more comprehensive depression questionnaire, or, preferably, by a structured interview.

## Conclusion

In this study, the personality trait harm avoidance was positively, and the character trait self-directedness negatively, associated with serum CRP levels in women. Previously published studies [76] have suggested that type D personality, described as a synergy between negative affectivity and social inhibition, is an important risk factor for morbidity and mortality in patients suffering from cardiovascular disease. In this respect it is tempting to suggest that one link between psychological distress and cardiovascular disease may be raised levels of CRP. The underlying mechanism behind the association between brain functions and peripheral low-grade inflammation, also demonstrated in ongoing depression, however remains unknown.

## Competing interests

The author(s) declare that they have no competing interests.

## Authors' contributions

SH performed the statistical analyses and has been involved in drafting the manuscript. FB, RR and GH are performing the longitudinal study with respect to women's health in which the subjects of this study are included. ML and HA performed the personality traits investigations and psychiatric evaluations. AE carried out the immunoassay, performed the analysis and interpretation of the statistical data together with SH and drafted the manuscript. All authors read, provided comments and approved the final manuscript.
